# Genome-Wide Association Study of Serum Creatinine Levels during Vancomycin Therapy

**DOI:** 10.1371/journal.pone.0127791

**Published:** 2015-06-01

**Authors:** Sara L. Van Driest, Tracy L. McGregor, Digna R. Velez Edwards, Ben R. Saville, Terrie E. Kitchner, Scott J. Hebbring, Murray Brilliant, Hayan Jouni, Iftikhar J. Kullo, C. Buddy Creech, Prince J. Kannankeril, Susan I. Vear, Kyle B. Brothers, Erica A. Bowton, Christian M. Shaffer, Neelam Patel, Jessica T. Delaney, Yuki Bradford, Sarah Wilson, Lana M. Olson, Dana C. Crawford, Amy L. Potts, Richard H. Ho, Dan M. Roden, Josh C. Denny

**Affiliations:** 1 Department of Pediatrics, Vanderbilt University School of Medicine and the Monroe Carell Jr. Children’s Hospital at Vanderbilt, Nashville, Tennessee, United States of America; 2 Center for Human Genetics Research, Vanderbilt University, Nashville, Tennessee, United States of America; 3 Department of Obstetrics and Gynecology, Vanderbilt University, Nashville, Tennessee, United States of America; 4 Department of Biostatistics, Vanderbilt University, Nashville, Tennessee, United States of America; 5 Center for Human Genetics, Marshfield Clinic, Marshfield, Wisconsin, United States of America; 6 Division of Cardiovascular Diseases, Department of Medicine, Mayo Clinic College of Medicine, Rochester, Minnesota, United States of America; 7 Institute for Clinical and Translational Research, Vanderbilt University, Nashville, Tennessee, United States of America; 8 Department of Pharmacology, Vanderbilt University, Nashville, Tennessee, United States of America; 9 School of Medicine, Vanderbilt University, Nashville, Tennessee, United States of America; 10 Department of Medicine, Vanderbilt University Medical Center, Nashville, Tennessee, United States of America; 11 Department of Pharmaceutical Services, Vanderbilt University, Nashville, Tennessee, United States of America; 12 Department of Biomedical Informatics, Vanderbilt University, Nashville, Tennessee, United States of America; The University of Texas Health Science Center (UTHSCSA), UNITED STATES

## Abstract

Vancomycin, a commonly used antibiotic, can be nephrotoxic. Known risk factors such as age, creatinine clearance, vancomycin dose / dosing interval, and concurrent nephrotoxic medications fail to accurately predict nephrotoxicity. To identify potential genomic risk factors, we performed a genome-wide association study (GWAS) of serum creatinine levels while on vancomycin in 489 European American individuals and validated findings in three independent cohorts totaling 439 European American individuals. In primary analyses, the chromosome 6q22.31 locus was associated with increased serum creatinine levels while on vancomycin therapy (most significant variant rs2789047, risk allele A, β = -0.06, p = 1.1 x 10^-7^). SNPs in this region had consistent directions of effect in the validation cohorts, with a meta-p of 1.1 x 10^-7^. Variation in this region on chromosome 6, which includes the genes *TBC1D32/C6orf170* and *GJA1* (encoding connexin43), may modulate risk of vancomycin-induced kidney injury.

## Introduction

Vancomycin is a commonly used glycopeptide antibiotic with activity against gram positive bacteria, including methicillin-resistant *Staphylococcus aureus*.[1] Though vancomycin is not metabolized, but excreted unchanged by the kidney, vancomycin serum concentrations vary widely among individuals, even after adjustment for renal function, indicating variability in individual pharmacokinetics.[2,3] Current guidelines recommend therapeutic vancomycin drug monitoring via measurement of trough concentrations and weight-based vancomycin dosing.[4]

One adverse event associated with vancomycin use is nephrotoxicity, occurring in 3–19% of patients treated with conventional drug doses.[5,6] High dose therapy is associated with higher incidence of nephrotoxicity; vancomycin regimens of more than 4 g/day or targeting trough concentrations over 20 mcg/mL result in renal toxicity in 30–40% of patients.[7] Additional risk factors for vancomycin nephrotoxicity include elevated creatinine at baseline, concomitant nephrotoxic agents, age, and intensive care unit admission.[6] While the effects on the kidneys are reversible, renal injury during vancomycin therapy has the potential to further exacerbate drug toxicities, as renal elimination of this and other drugs may be impaired.[7]

Given the variability of vancomycin kinetics, frequency of nephrotoxicity, and potential clinical impact of variability in each, we sought to identify genomic loci associated with these outcomes. In a genome-wide association study (GWAS) of a retrospective cohort of individuals treated with vancomycin, we examined associations in the primary cohort of 489 individuals with peak creatinine level during the first two weeks of vancomycin therapy, as well as vancomycin trough levels and calculated renal elimination rate constant (Ke). Associations were validated via analysis and meta-analysis of data from independent cohorts of 343 and 59 individuals from two other medical centers, and an additional 37 individuals from the primary site. All three sites participate in the Electronic Medical Records and Genomics (eMERGE) Network and utilized electronic medical record (EMR) data and biobank specimens to assemble the investigated cohorts.[8]

## Methods

### Ethics Statement

The primary cohort for this study was derived from BioVU, Vanderbilt’s repository linking DNA from remnant clinical blood samples to de-identified EMR data.[9,10] This resource has been approved as non-human subjects research by Vanderbilt's local Institutional Review Board and the federal Office of Human Research Protections (OHRP). This study was also reviewed by the Vanderbilt Institutional Review Board and determined to be non-human subjects research. Individuals at validation sites provided written consent as part of the DNA biobank at each site.[11] This study was approved by the Institutional Review Board at both validation sites (Marshfield Clinic and Mayo Clinic).

### Primary Cohort Identification

The primary cohort included only European American individuals, determined by genotype as described below, 18 years of age or older in BioVU with documentation of a vancomycin trough level after the third dose, the associated dose and dosing interval, and serum creatinine measurements. Exclusion criteria included: no vancomycin trough obtained after the third dose (reflecting steady state); dialysis therapy, extracorporeal membrane oxygenation, or heart transplantation prior to or during vancomycin therapy, identified by current procedural terminology (CPT) codes and manual review; and documentation of multiple dosing regimens of vancomycin prior to trough which were unable to be resolved by manual review.

### Outcome and Covariate Definitions for Peak Serum Creatinine

Peak serum creatinine was defined as the highest creatinine value obtained two dosing intervals after initiation of vancomycin through two weeks after the start of therapy (S1 Fig). The *a priori* selected covariates for the outcome of peak creatinine were sex, age at time of vancomycin therapy, height, weight, vancomycin dose and dosing interval, vancomycin trough, and two serum creatinine values (baseline and creatinine at vancomycin start, S1 Fig). Baseline creatinine was defined as the lowest creatinine value measured from one month before the start of vancomycin through the third dose and was included to reflect optimal renal function prior to or at the start of illness. The creatinine at vancomycin start, included to reflect renal function at the time of initiation of therapy, was defined as the creatinine value closest in time to the start of vancomycin therapy with preference for values obtained in the 24 hours prior to the start of therapy, then in the first 24 hours of therapy, then up to 30 days prior to the start of therapy. The frequency of acute kidney injury (AKI), defined as a rise in the serum creatinine of 0.3mg/dL or a 1.5-fold increase in serum creatinine from the baseline to peak value,[12] was assessed, and this dichotomous definition of AKI was evaluated as a secondary outcome.

Because concomitant medications, including diuretic and nephrotoxic medications, affect renal function, vancomycin excretion, and serum creatinine levels, all medication orders for loop diuretic medications (furosemide, bumetanide, torsemide, and ethacrynate) within 72 hours before vancomycin trough measurement were extracted from EMR data. Using drug, dose, frequency and route data, diuretic exposures were converted to IV furosemide equivalents given per 24 hours using the following conversions: 1mg oral furosemide = 0.5mg; 1mg oral or IV bumetanide = 40mg; 1mg oral or IV torsemide = 2mg; and 1mg oral or IV ethacrynate = 1mg. For non-loop diuretics, medication data were extracted to determine the total number of different non-loop diuretics given 72 hours prior to the vancomycin trough measurement; specific medications identified in these cohorts were eplerenone, hydrochlorothiazide, mannitol, metolazone, spironolactone, and triamterene. The number of different nephrotoxic medications given to each patient in the 72 hours prior to the vancomycin trough was tallied, excluding those given via topical, ophthalmic or otic routes of administration (listed in S1 Table). Contrast agents were restricted to those administered intravenously. All “PRN” or “as needed” orders were manually reviewed, and included in the tally only if the EMR included evidence that the patient actually received the nephrotoxic medication.

### Outcome and Covariate Definitions for Vancomycin Trough and Ke

Vancomycin trough was defined as the first vancomycin trough documented in the EMR after at least three doses of vancomycin were given. Ke for each individual was calculated using the formula Ke = -ln[(Trough + [dose/(0.65 x weight)])/Trough]/(dosing interval—infusion time). Covariates included in the analysis of vancomycin trough were age, sex, height, weight, body surface area, vancomycin dose and dosing interval, creatinine at vancomycin start, and concomitant diuretic and nephrotoxic drugs, as defined above. For the analysis of calculated vancomycin Ke, weight, vancomycin dose, and vancomycin dosing interval were excluded as covariates, as they are used in the calculation of Ke. For these two outcomes, multiple imputation was used for missing covariate data points.

### Data Extraction and Validation

All outcome and covariate data were initially extracted from the BioVU repository using automated strategies. After data extraction, a portion of all records was manually reviewed to confirm data accuracy. Manual review included appropriate application of exclusion criteria (e.g. all individuals with any history of a dialysis CPT code were reviewed to ensure dialysis was not initiated during vancomycin therapy), confirmation of dosing data (e.g. all individuals with clinical orders indicating different dosage or interval for vancomycin therapy were reviewed to determine the dose relevant to the trough), and review of outliers (all values more than two standard deviations from the mean for age, height, weight, body surface area, vancomycin dose, vancomycin interval, creatinine, vancomycin trough, and all concomitant medication exposures). Any inaccuracies were manually corrected. All data were stored using the research database tool REDCap.[13]

### Primary Genotyping and Quality Control

DNA samples from the primary cohort were genotyped using the Omni1-Quad BeadChip array (Illumina, San Diego, CA). Quality control of genotyping included exclusion of samples with discordant gender compared to biobank records, genotyping efficiency (GE) < 98%, cryptic relatedness (two pairs of half-siblings identified, individuals with lower genotyping efficiency excluded from analysis), and duplicate samples. Single nucleotide polymorphisms (SNPs) were excluded from analysis if minor allele frequency was < 5%, GE < 98% across all samples, discordant genotypes called in duplicate samples, Mendelian errors identified among HapMap trios, or Hardy-Weinberg Equilibrium p-value was > 0.001.

### Primary Analysis and Imputation

All cohorts with genome-wide genotyping were restricted to those with European American ancestry indicated by analysis of ancestry informative markers using STRUCTURE.[14] Ancestry distribution for the primary cohort before and after elimination of individuals of non-European American ancestry is shown in S2 Fig. Association analysis of the primary genotyped dataset to each outcome was completed using PLINK v1.07.[15] Linear regression assuming an additive genetic model was used to test for single SNP association adjusting for covariates defined above. Individuals missing data for the outcome variable or any covariate data were excluded from the peak creatinine analysis. Due to leftward skew of the outcome variables, peak creatinine, vancomycin trough, and Ke values were log transformed to satisfy normality assumptions. Restricted cubic splines were initially considered to allow non-linear associations between the respective outcomes and continuous covariates, including age, height, weight, body surface area, creatinine, and vancomycin trough. For the outcome of peak creatinine, these were reduced to linear terms for easier interpretation after observing no evidence of non-linearity. After primary association analysis, individual chromosomes with the most significant loci for each outcome were imputed to determine additional SNPs using IMPUTE v2.2.2 and all 1000 Genomes populations as a reference set October 2012 build.[16]

### Validation Cohort Analyses

External validation cohorts were assembled using European American individuals from biobanks at the Marshfield Clinic in Marshfield, WI (343 individuals from the Personalized Medicine Research Project who were genotyped for this analysis) and the Mayo Clinic College of Medicine in Rochester, MN (59 individuals with previous genotype data available from prior analyses). An additional 37 European American individuals from the primary site, not included in the discovery analyses and genotyped as part of the Vanderbilt Genome-Electronic Records (VGER) project, were also identified. Inclusion criteria, exclusion criteria, outcome variables, covariates, and data validation procedures for these cohorts were the same as those for the primary cohort.

The most highly associated SNPs in each region identified in the analysis of genotyped data and imputed data as well as tag SNPs for each region identified were genotyped using Sequenom MassARRAY (Sequenom Inc., San Diego, CA) in the 343 DNA samples from the Marshfield Clinic cohort. Linear regression analyses to determine the association of each genotyped SNP to the outcomes of peak serum creatinine, vancomycin trough, and vancomycin Ke were performed with SNPTest v2.4.1.[17] An additional 96 samples from the Mayo Clinic and BioVU cohorts were identified with genotyping completed on the Human660W-Quad Bead Chip (Illumina, San Diego, CA). Single locus association analyses across primary and all three validation cohorts were further analyzed together with fixed-effects meta-analyses using METAL software.[18] The effective number of independent tests was calculated using SimpleM.[19,20]

## Results

### Primary Cohort

**Fig 1:**
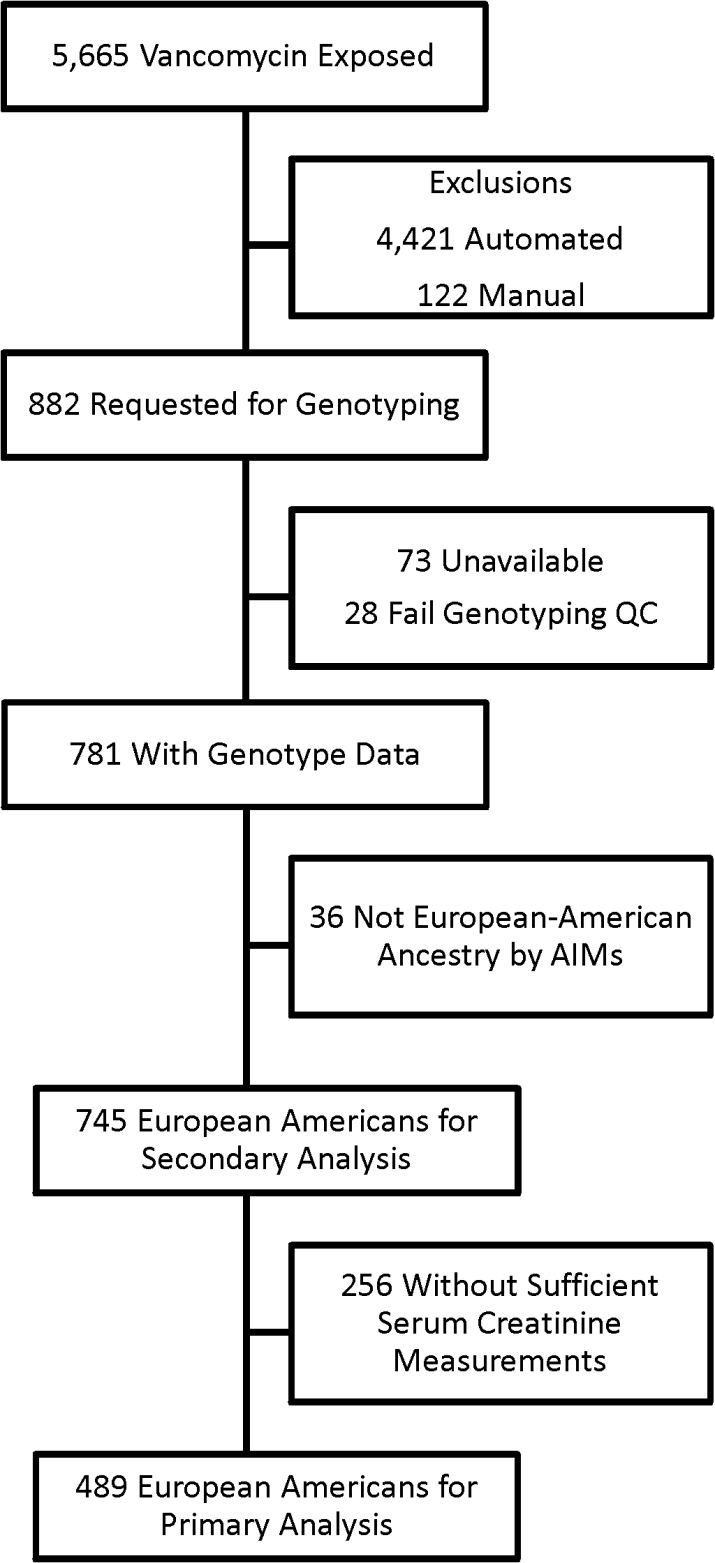
Identification of primary cohort. Electronic medical records data were searched to identify 5,665 individuals exposed to vancomycin. Automated and manual algorithms were used to determine if each satisfied inclusion / exclusion criteria, as described in the methods, resulting in 882 confirmed cases. After exclusion of those without DNA, those who failed quality control (QC), and those of non-European-American ancestry, 745 individuals remained. Of those, 489 had serum creatinine measurements for the primary analysis.

We identified a primary cohort of 882 individuals in the Vanderbilt biorepository, BioVU (Fig 1). After excluding individuals with no DNA sample available (N = 73), those who failed genotyping quality control (N = 28), and non-European-Americans (N = 36), 745 individuals remained. Of those, 256 were missing data for one or more of the requisite serum creatinine measurements, resulting in a primary cohort of 489 European American individuals for the analysis of peak creatinine while on vancomycin (Table 1). The median age was 55 years, and 59% of the cohort was male. Most individuals were treated with 1000 mg of vancomycin every 12 hours and had vancomycin trough measurements in therapeutic range (interquartile range 7–16 mcg/mL). The majority of patients were not exposed to diuretic medications, but did receive one or more nephrotoxic drugs concomitant to vancomycin therapy. Peak serum creatinine measurements were higher than creatinine measurements at baseline or at vancomycin start (see methods for definitions). Of the 489 individuals, 188 (38%) had serum creatinine measurements which met AKI criteria.

**Table 1:** Cohort demographics for primary and validation cohorts investigated by genome-wide association for the outcome of peak serum creatinine during vancomycin therapy.

	Primary Cohort	Marshfield Validation Cohort	Mayo Validation Cohort	VGER Validation Cohort
	(N = 489)	(N = 343)	(N = 59)	(N = 37)
Age (years)*	55 (43–65)	64 (53–75)	70 (58–75)	54 (45–66)
Male^	289 (59%)	197 (57%)	39 (66%)	17 (46%)
Weight (kg)*	81 (66–97)	86 (68–103)	88 (74–100)	82 (68–96)
Height (m)*	173 (165–180)	168 (161–177)	170 (162–182)	175 (165–180)
Vancomycin Dose (mg)*	1000 (1000–1000)	1200 (1000–1500)	1400 (1100–1500)	1000 (1000–1000)
Vancomycin Dosing Interval (h)*	12 (12–12)	12 (12–24)	12 (12–24)	12 (8–12)
Vancomycin Trough (mcg/mL)*	10 (7–16)	11 (8–16)	12 (10–15)	12 (7–17)
Baseline Creatinine (mg/dL)*	0.7 (0.6–0.9)	0.8 (0.6–1.0)	0.9 (0.8–1.0)	0.8 (0.6–1.1)
Creatinine at Vancomycin Start (mg/dL)*	0.9 (0.7–1.1)	0.9 (0.7–1.1)	1.0 (0.9–1.2)	0.8 (0.7–1.0)
24-hour Loop Diuretic Dose (mg)*	0 (0–10)	0 (0–0)	0 (0–20)	0 (0–0)
Loop Diuretic Exposure^	134 (27%)	33 (10%)	23 (39%)	2 (5%)
Number of Non-loop Diuretics^				
0	423 (87%)	314 (92%)	41 (69%)	30 (81%)
1 or more	66 (13%)	29 (8%)	18 (31%)	7 (19%)
Number of Nephrotoxic Drugs^				
0	164 (34%)	225 (66%)	8 (14%)	16 (43%)
1	190 (39%)	50 (15%)	24 (41%)	10 (27%)
2 or more	135 (28%)	68 (20%)	27 (46%)	11 (30%)
Peak Creatinine (mg/dL)	1.0 (0.8–1.3)	1.0 (0.8–1.4)	1.1 (0.9–1.4)	0.9 (0.7–1.0)

*Median (interquartile range)

^N, %.

DNA samples were genotyped using the Illumina HumanOmni1-Quad platform, and SNPs were analyzed for quality control. Of the initial 1,138,747 variants, 19,793 SNPs were excluded due to genotyping efficiency less than 98%, 455 SNPs were excluded due to Mendelian errors, and 1,526 SNPs were excluded due to discordant calls across duplicate samples. After removal of SNPs with minor allele frequency < 0.05 and those out of Hardy-Weinberg Equilibrium, 711,284 SNPs remained for analysis. The effective number of independent tests calculated using SimpleM was 392,431, resulting in a calculated genome-wide alpha threshold of 1.27 x 10^–7^.[19,20]

**Fig 2:**
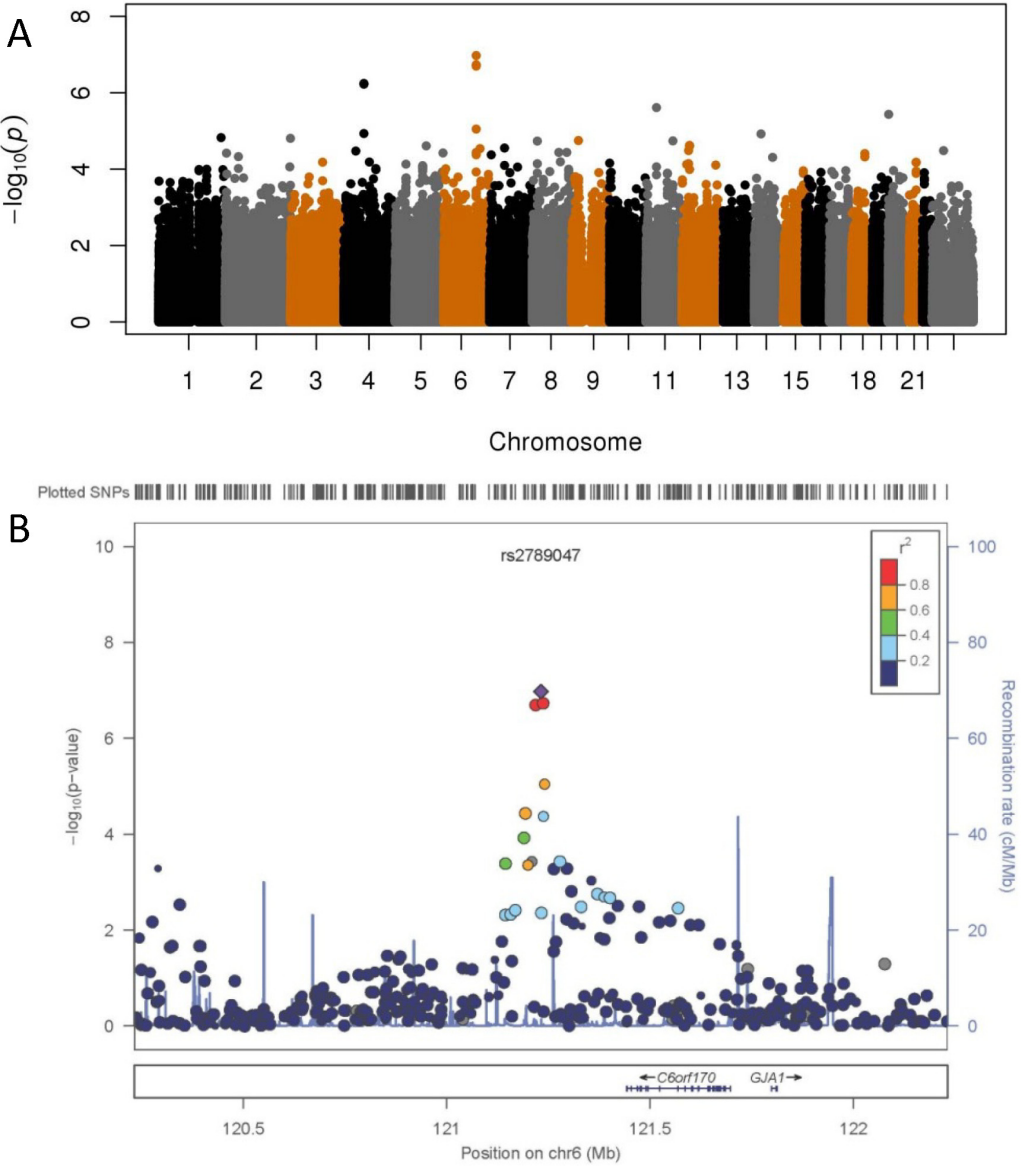
Association of genome-wide SNPs to peak creatinine while on vancomycin therapy in the primary cohort. A) Manhattan plot, where each dot represents a genotyped SNP, arranged along the x-axis by position of the SNP on each chromosome. The y-axis plots-log10(p-value) for the linear regression analysis of peak creatinine, adjusted for sex, age at time of vancomycin therapy, height, weight, vancomycin dose / dosing interval, vancomycin trough, and baseline serum creatinine measurements as described in the methods. B) LocusZoom plot of 6q22.31 locus, including genotyped and imputed SNPs. Each dot represents a SNP, arranged by position on chromosome 6 along the x-axis, and the color indicates degree of linkage disequilibrium with the index SNP, rs2789047. The left y-axis plots-log10(p-value) for each SNP. The blue line indicates estimated recombination rate, quantified on the right y-axis. Known genes in the region are indicted below the x-axis.

Linear regression analysis of genotyped SNPs, assuming an additive genetic model and adjusting for *a priori* determined covariates, identified the most significant SNP, rs2789047 (p = 1.1 x 10^–7^) at chromosome 6q22.31 (Fig 2). Analysis of all genotyped and imputed SNPs on chromosome 6 identified 6 SNPs with information quality score > 0.9 at the same locus with similar p-values (rs2817952 and rs2817953, p = 9.5 x 10^–7^; rs2817955, rs2817954, rs2817957 and rs2251428, p = 9.6 x 10^–7^). Analysis of AKI as a dichotomous outcome did not identify any SNPs with p-values < 1x10^-6^.

### Validation Cohorts and Meta-Analysis

We sought validation from three independent cohorts, including 343 individuals from the Marshfield Clinic, 59 individuals from Mayo Clinic, and 37 individuals from Vanderbilt not included in the primary analyses and genotyped as part of the Vanderbilt Genome-Electronic Records (VGER) project. All individuals were European Americans, ≥ 18 years of age, and received vancomycin during inpatient hospitalizations. Demographic characteristics and clinical covariates for the primary and validation cohorts are listed in Table 1. With the exception of the VGER cohort, the validation cohorts were older and had higher, less frequent vancomycin dosing than the primary cohort. Creatinine values were similar across cohorts, with peak creatinine values higher than those defined as baseline or at vancomycin start. Diuretic and nephrotoxic medication exposures varied in frequency across cohorts, with the Mayo cohort having higher frequencies of exposure to these medications.

A total of 65 SNPs with minor allele frequency > 1% from the chromosome 6 locus, including the most statistically significant SNPs and additional SNPs tagging known variants in the region, were genotyped in the validation set of 343 Marshfield samples, resulting in 12 SNPs with p-values for association with peak creatinine < 0.05 in adjusted analysis (S2 Table). Meta-analysis of the primary and all validation cohorts for the SNPs in this region represented in the majority of datasets, either as genotyped or imputed SNPs, revealed 3 SNPs with p-values < 3 x 10^–7^ with consistent direction of effect in all but the supplemental VGER cohort (Table 2); the p-value for the most statistically significant SNP in the meta-analysis (rs2789047, p = 1.1 x 10^–7^) exceeds the calculated genome-wide alpha threshold of 1.27 x 10^–7^. The three most significantly associated SNPs have identical effect sizes (β = -0.053) and are in strong LD, with r^2^ > 0.92 for all pairwise calculations of LD in the primary cohort. Genes in this region include *TBC1D32/C6orf170*, located ~211kb downstream and *GJA1*, encoding connexin43, located ~567kb downstream (Fig 2).

**Table 2:** Meta-analysis results for chromosome 6 SNP associations to peak creatinine during vancomycin therapy.

			Primary Cohort (N = 489)			Marshfield Validation (N = 343)		Mayo Validation (N = 59)		VGER Validation (N = 37)		Meta-Analysis (Primary + Validation)	
SNP	Position	Risk Allele	RAF	β (SE)	P	β (SE)	P	β (SE)	P	β (SE)	P	β (SE)	Meta-P
rs2789047	121231501	A	0.28	-0.06 (0.01)	1.1x10^-7^	-0.02 (0.04)	0.57	-0.001 (0.07)	0.99	0.09 (0.37)	0.81	-0.05 (0.01)	1.1x10^-7^
rs12527161	121236583	T	0.27	-0.06 (0.01)	1.8x10^-7^	-0.02 (0.04)	0.63	-0.02 (0.07)	0.76	0.09 (0.37)	0.81	-0.05 (0.01)	1.8x10^-7^
rs9320773	121218196	A	0.27	-0.06 (0.01)	2.0x10^-7^	-0.01 (0.04)	0.82	-0.04 (0.08)	0.62	0.001 (0.34)	1.0	-0.05 (0.01)	2.3x10^-7^
rs1886249	121791852	c	0.14	-0.02 (0.01)	0.13	-0.04 (0.04)	0.35	-0.03 (0.08)	0.74	0.30 (0.43)	0.50	-0.023 (0.01)	0.081
rs3805787	121800606	c	0.23	-0.01 (0.01)	0.43	-0.03 (0.04)	0.37	-0.04 (0.07)	0.59	0.26 (0.46)	0.58	-0.012 (0.01)	0.28
rs17083553*	121775368	T	0.19	0.01 (0.01)	0.65	0.07 (0.05)	0.12	0.01 (0.07)	0.99	-0.37 (0.44)	0.41	0.009 (0.01)	0.413
rs6932373*	121832689	A	0.38	0.002 (0.01)	0.87	-0.04 (0.03)	0.27	-0.09 (0.07)	0.19	0.08 (0.23)	0.74	-0.003 (0.01)	0.752
rs2389541	121815698	c	0.31	0.01 (0.01)	0.31	-0.07 (0.04)	0.05	-0.09 (0.07)	0.20	0.10 (0.26)	0.70	0.002 (0.01)	0.805
rs17083598	121788169	g	0.06	0.01 (0.02)	0.75	-0.09 (0.06)	0.18	-0.08 (0.16)	0.62	1.01 (0.63)	0.12	-0.002 (0.02)	0.902

RAF—Risk Allele Frequency; SE—Standard Error.

*—Imputed SNP in Mayo and VGER cohorts.

### Analysis of Vancomycin Trough and Ke

No individuals in the primary cohort were missing outcome data for the analysis of vancomycin trough or calculated Ke, allowing analysis of 745 individuals (S3 Table). In the analysis of the 711,284 genotyped SNPs in the primary cohort, both vancomycin trough and Ke analyses identified SNPs at chromosome 1q41 (S3 and S4 Figs). The most significantly associated SNP on chromosome 1 was rs10495197 (p = 4.5 x 10^–7^ for vancomycin trough and 2.4 x 10^–6^ for Ke). Imputation of chromosome 1 and analysis of imputed genotypes identified 13 additional SNPs at this locus associated with vancomycin trough and Ke with smaller p-values. Analysis of 27 SNPs on chromosome 1 genotyped in the Marshfield cohort, including SNPs with smallest p-values from the primary analysis and tag SNPs for known variants in these regions, found a minimum p-value of 0.13 for the outcome of vancomycin trough (S4 Table). Effect sizes and directions of effect were variable for chromosome 1 SNPs between the primary and replication cohorts.

Chromosome locus 5q14.3 was also associated with these outcomes (S3 and S4 Figs). On chromosome 5, the most significant p-values for genotyped SNPs were obtained for rs12518285 (p = 9.2 x 10^–6^ for vancomycin trough and 1.8 x 10^–7^ for Ke), and 5 imputed SNPs had smaller p-values. Analysis of 5 associated and tag SNPs on chromosome 5 in the Marshfield cohort found a minimum p-value of 0.16 (S4 Table). Meta-analysis of the primary and validation cohorts using both genotyped and imputed SNPs, for the outcome of vancomycin trough revealed the lowest p-value 6.0 x 10^–5^ (rs12518285, on chromosome 5, S4 Table). Effect sizes and directions of effect were consistent for chromosome 5 SNPs between the primary and replication cohorts. The nearest known gene adjacent to the chromosome 5 locus is *EDIL3*, 557kb upstream.

Of note, SNPs at the chromosome 6 locus identified in the analysis of peak creatinine were not significantly associated with vancomycin trough or Ke. The most significant association in this region for both outcomes was for rs7748279 (p = 0.03 and 0.12 for association with vancomycin trough and Ke, respectively).

## Discussion

We utilized banked DNA and de-identified EMR data to assemble primary and validation cohorts to identify genetic variants associated with peak serum creatinine while on vancomycin, vancomycin trough concentration, and vancomycin Ke. The GWAS and meta-analysis identified a potential association of the chromosome 6q22.31 locus to peak creatinine during vancomycin therapy. Neither the primary analysis nor the meta-analysis resulted in p-values below 5 x 10^–8^, the typical threshold value for genome-wide significance, which may be due to the modest sample size of the primary and validation cohorts for this study design. However, p-values below the alpha threshold of 1.27x10^-7^ were found in the primary cohort and the meta-analysis across cohorts of peak creatinine levels while on vancomycin.

Vancomycin requires intravenous administration for systemic delivery due to poor oral bioavailability, so nearly all patients treated with vancomycin receive this drug during an inpatient hospitalization. At the primary institution and the validation sites, EMRs retain the patient data, laboratory values and medication exposures necessary for this study of vancomycin. BioVU, the Vanderbilt biorepository linked to de-identified EMR data, has DNA samples and clinical data available for over 180,000 individuals. We identified over 5,600 vancomycin-exposed individuals. However, the number of individuals with sufficient clinical documentation of outcomes (including multiple measured serum creatinine values and measurement of the vancomycin trough after at least 3 doses had been given) and covariates (including vancomycin dosing data) was much smaller. Cohort size was further reduced by the necessary step of restricting the analysis by ancestry. Despite the inclusion of a total of only 928 individuals from all sites for the primary outcome of peak serum creatinine while on vancomycin, the 6q22.31 locus was associated with a meta-p-value of 1.1 x 10^–7^.

The SNP with the strongest evidence of association with peak serum creatinine while on vancomycin (rs2789047) is near two known genes. The first, *TBC1D32/C6orf170*, encodes a ciliary protein and has been associated with oro-facial-digital syndrome type IX.[21,22] There are no reported associations of this protein to renal function. The second gene in this region is *GJA1*, which encodes connexin43, a gap junction protein expressed in renal proximal tubules that has been previously associated with renal injury.[23–25] In rodents, podocyte injury and chronic kidney disease lead to increased expression of connexin43.[23,24] In human proximal tubule epithelial cell cultures, aminoglycoside-sensitive cell lines have increased levels of expression of connexin43.[25] Connexin43 overexpression sensitized cells to aminoglycoside-induced injury, and siRNA inhibition of connexin43 expression attenuated cellular injury, as did functional inhibition.[25] We speculate that genetic variation in the locus we identified may affect connexin43 expression or function in humans and in turn play a role in nephrotoxicity from vancomycin. This region was not associated with vancomycin trough or Ke, suggesting that it may be an independent risk factor for kidney injury. Prior GWAS analyses in multiple populations have not identified this locus in association with chronic kidney disease,[26–30] indicating that this finding may be specific for nephrotoxicity in the setting of vancomycin exposure. Further experimental data are required to determine the specific role, if any, connexin43 may play in vancomycin-associated renal injury.

Analysis of the secondary outcomes (vancomycin trough and vancomycin Ke) failed to identify loci with p-values exceeding our alpha threshold in the primary or meta-analyses. The most strongly associated loci on chromosome 1 and chromosome 5 weakly associated with both outcomes, as expected since Ke was calculated from vancomycin trough, dose, and patient weight. Although a genetic association to variation in vancomycin clearance was not identified, our study demonstrates the use of EMR data to explore complex phenotypes such as pharmacokinetics.

Conclusions to be drawn from this initial GWAS are limited by issues common across the use of contemporary genomic approaches to study variability in drug action: modest cohort size, small effect size, and the selected nature of the individuals represented in these cohorts (i.e., those receiving vancomycin). The requirement for serum creatinine and vancomycin trough measurements in the retrospective cohorts may have resulted in over-representation of high-risk individuals. This selection bias for sicker patients is a major limitation to this study and impacts the generalizability of our findings. In addition, because the full spectrum of clinical outcomes may not be well-represented in our cohorts, the full effects of genetic variation may not be apparent from our data. However, many of these high-risk individuals did not develop renal injury (as defined by elevated serum creatinine) providing a cohort sufficient to identify the chromosome 6 locus for further study. Our analysis of AKI as a dichotomous outcome failed to identify any SNPs with significant association, likely due to loss of statistical power with a dichotomous outcome and the lack of specificity for true renal injury that may be inherent to the currently accepted creatinine thresholds for AKI.

Additional study of variants in the connexin43 gene and surrounding regulatory DNA will be required to test the hypothesis that genetic variation at this locus may influence susceptibility to vancomycin-associated renal toxicity and assess the potential clinical impact of this association for patients treated with vancomycin and other renally eliminated nephrotoxic medications. If genetic markers for nephrotoxic risk with clinically significant effect size are identified, pre-treatment screening may be employed to identify patients who merit close monitoring or modifications to their vancomycin dose. In addition, as the cellular mechanisms for vancomycin elimination and associated nephrotoxicity are unknown, further exploration of the role of connexin43 may lead to novel therapeutic interventions to prevent vancomycin-associated kidney injury.

## Supporting Information

S1 Fig Definition of serum creatinine measurements for study. Three serum creatinine measurements were defined as depicted.
(TIF)

S2 Fig Ancestry Distribution by Principal Components. First and second principal components for study samples (Vanc) and HapMap samples (CEU, YRI, MEX, ASW, CHB_JPT) before (A) and after (B) restricting to European American individuals based on STRUCTURE analysis.
(TIF)

S3 Fig Association of genome-wide SNPs to vancomycin trough and Ke in the primary cohort. Each dot represents a genotyped SNP, arranged along the x-axis by position of the SNP on each chromosome. The y-axis plots −log10(p-value) for the linear regression analysis of each SNP to the outcome of interest, adjusted for the covariates defined in the methods. A) Manhattan plot of association p-values with log-transformed vancomycin trough levels. B) Manhattan plot of association p-values with log-transformed vancomycin Ke, the renal elimination rate constant.
(TIF)

S4 Fig QQ Plots. Shown are the expected (x-axis) vs. observed (y-axis) association p-values for A) peak creatinine while on vancomycin therapy, B) vancomycin trough levels, and C) vancomycin Ke.
(TIFF)

S1 Table Nephrotoxic medications. (DOCX)

S2 Table Association of genotyped candidate chromosome 6 SNPs to peak creatinine in Marshfield validation cohort of 343 individuals. (DOCX)

S3 Table Primary cohort demographics for the outcomes of vancomycin trough and vancomycin renal elimination rate constant, Ke. (DOCX)

S4 Table Results for chromosome 1 and 5 SNP association with vancomycin trough in primary and validation cohorts. (DOCX)
