# Supplementary figures and images for: Genome-Wide Association Study of Serum Creatinine Levels during Vancomycin Therapy
Source: PLoS One. 2015 Jun 1;10(6):e0127791. doi: 10.1371/journal.pone.0127791 (PMC4452656; doi:10.1371/journal.pone.0127791)

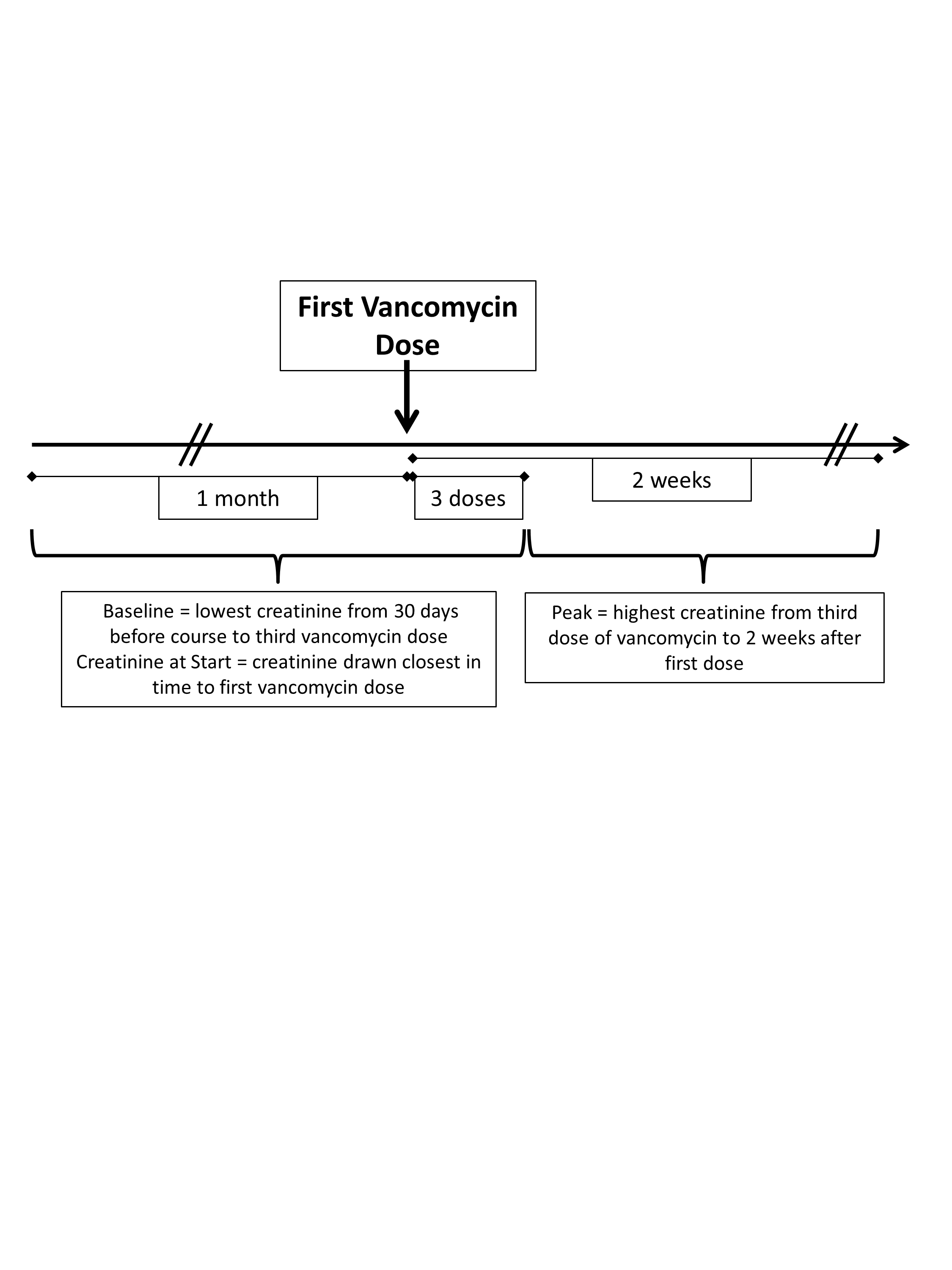

Supplement: S1 Fig — Three serum creatinine measurements were defined as depicted. (TIF) [file pone.0127791.s001.TIF]

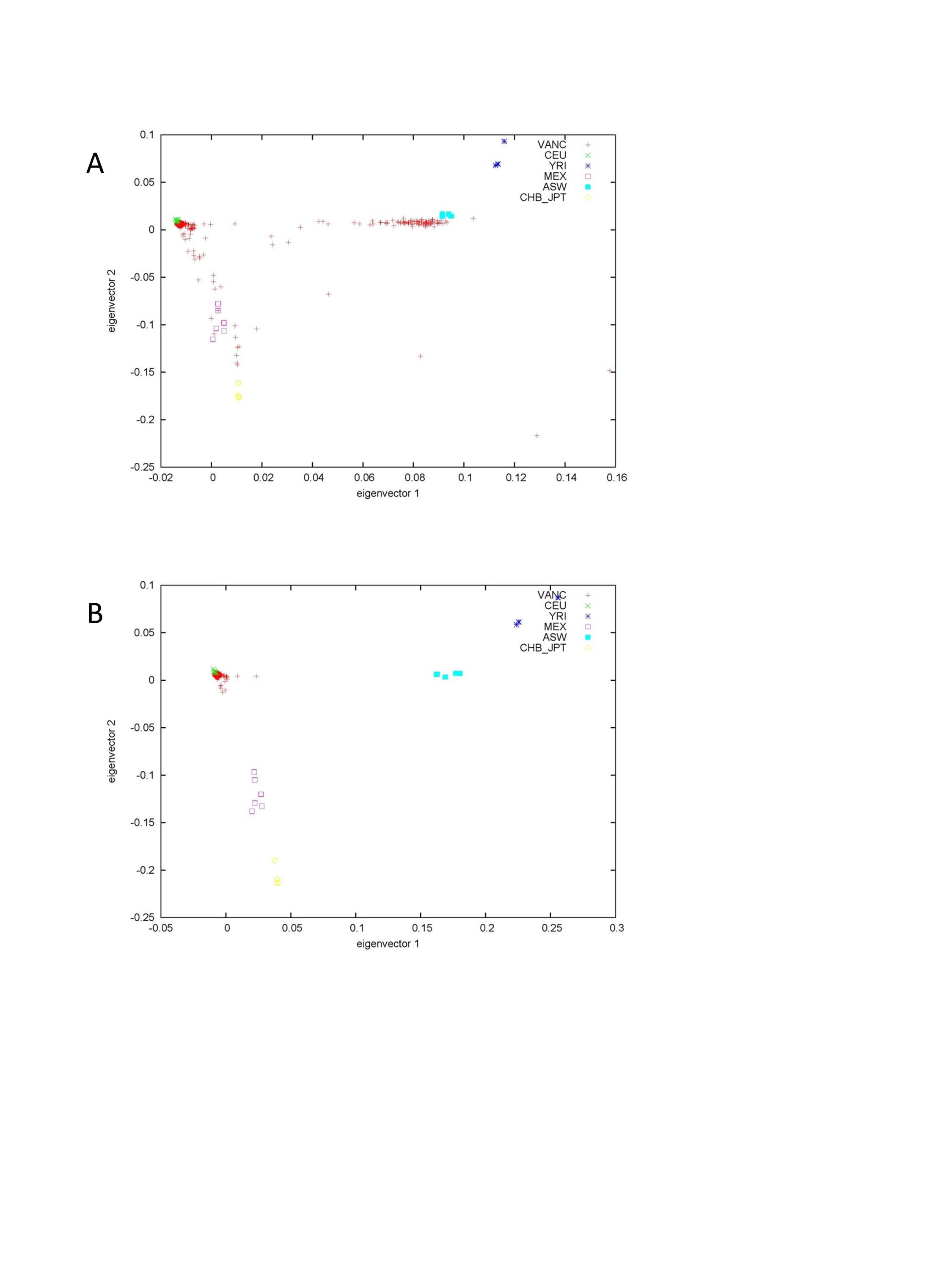

Supplement: S2 Fig — First and second principal components for study samples (Vanc) and HapMap samples (CEU, YRI, MEX, ASW, CHB_JPT) before (A) and after (B) restricting to European American individuals based on STRUCTURE analysis. (TIF) [file pone.0127791.s002.TIF]

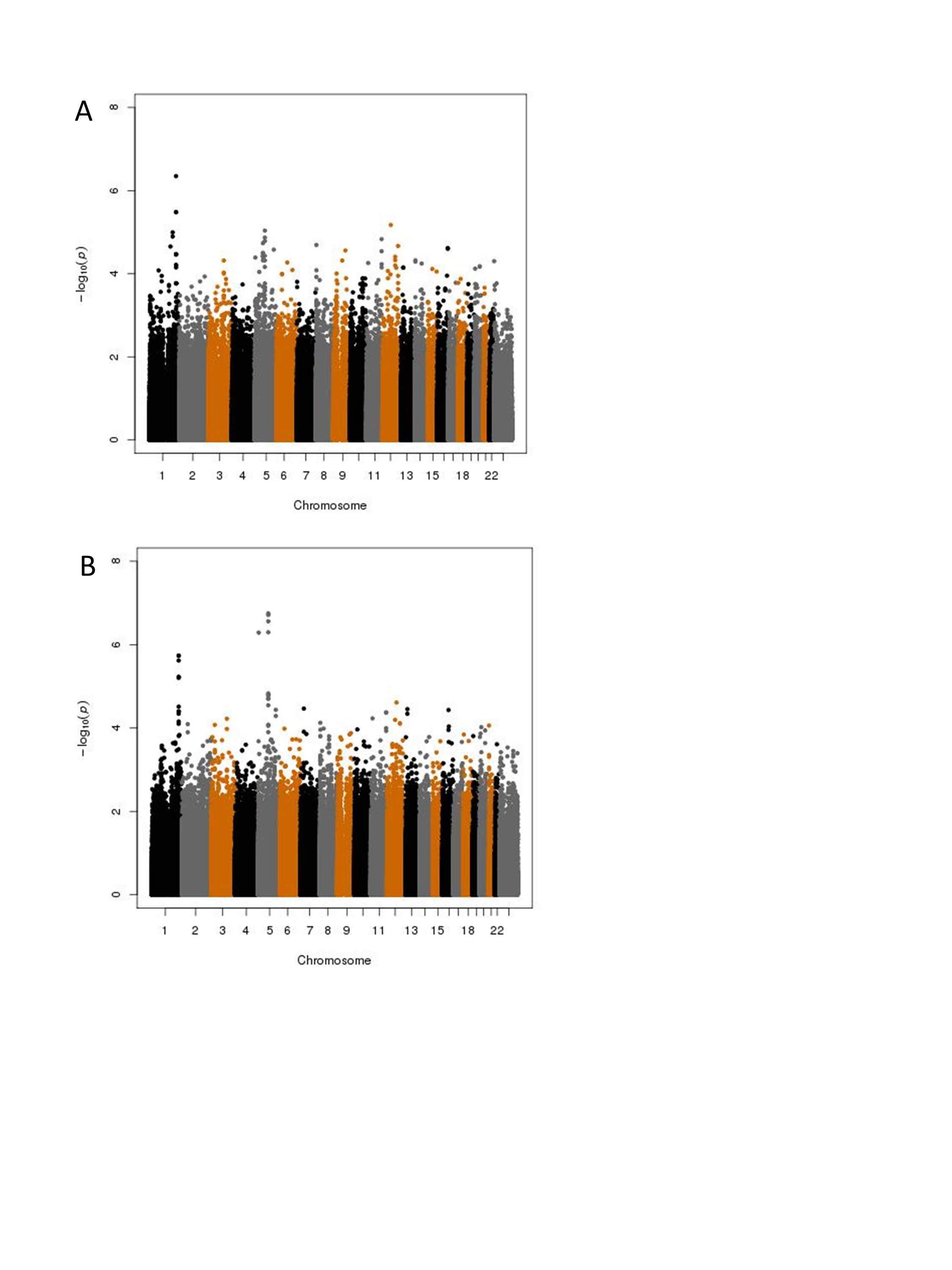

Supplement: S3 Fig — Each dot represents a genotyped SNP, arranged along the x-axis by position of the SNP on each chromosome. The y-axis plots −log10(p-value) for the linear regression analysis of each SNP to the outcome of interest, adjusted for the covariates defined in the methods. A) Manhattan plot of association p-values with log-transformed vancomycin trough levels. B) Manhattan plot of association p-values with log-transformed vancomycin Ke, the renal elimination rate constant. (TIF) [file pone.0127791.s003.TIF]

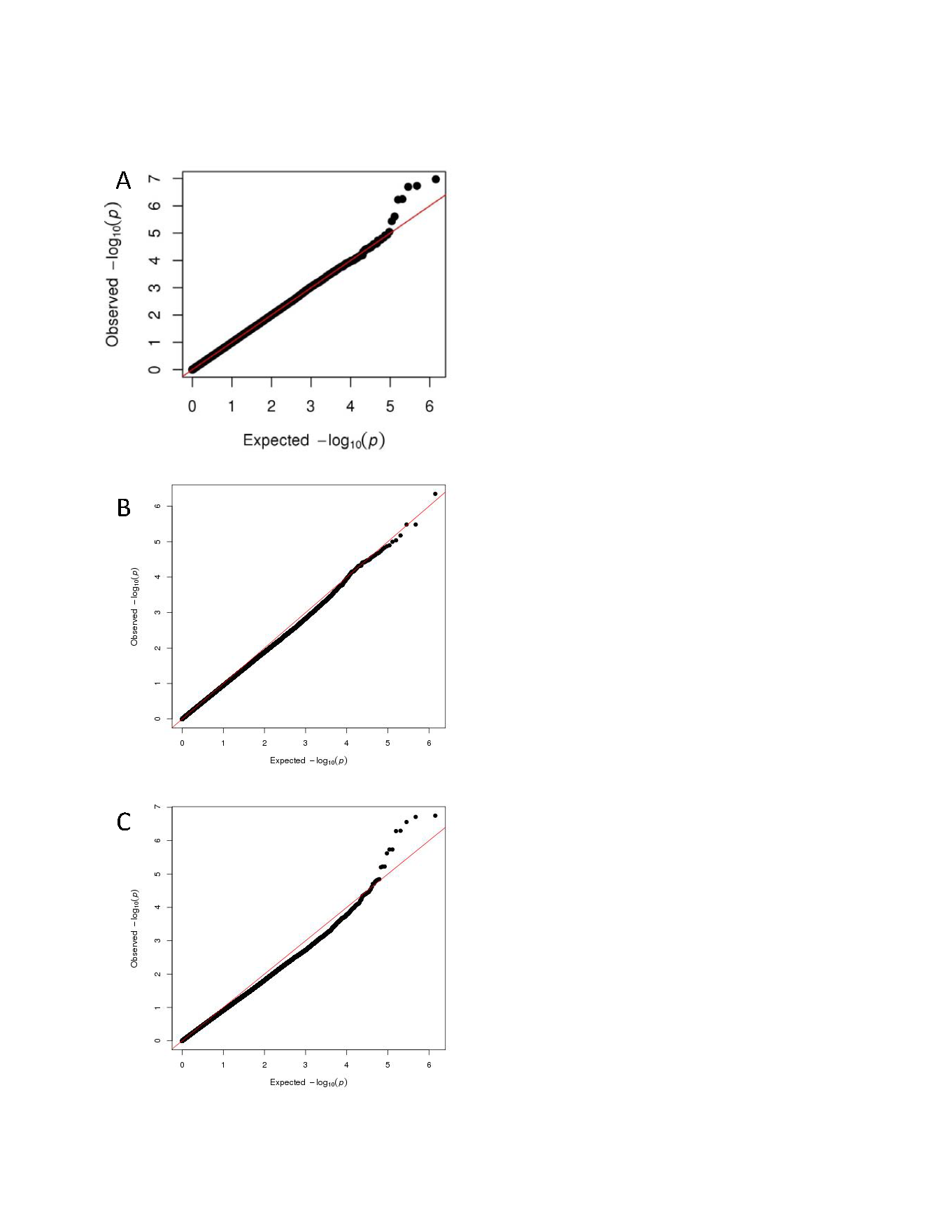

Supplement: S4 Fig — Shown are the expected (x-axis) vs. observed (y-axis) association p-values for A) peak creatinine while on vancomycin therapy, B) vancomycin trough levels, and C) vancomycin Ke. (TIFF) [file pone.0127791.s004.tiff]
