# Supplementary material for: Genome-Wide Association Study of Serum Creatinine Levels during Vancomycin Therapy
Source: PLoS One. 2015 Jun 1;10(6):e0127791. doi: 10.1371/journal.pone.0127791 (PMC4452656; doi:10.1371/journal.pone.0127791)
Supplement: S1 Table — (DOCX) [file pone.0127791.s005.docx]

**S1 Table.** **Nephrotoxic medications.**

| **Category** | **Drug name in electronic medical record** | **Category** | **Drug name in electronic medical record** |
| --- | --- | --- | --- |
| Angiotensin converting enzyme inhibitors | Lisinopril | Foscarnet | Foscarnet |
|  | Enalapril | Ganciclovir | Ganciclovir |
|  | Captopril | Intravenous immunoglobulin | Immune globulin IV |
|  | Altace |  | IVIG |
|  | Ramipril | Methotrexate | Methotrexate |
|  | Enalaprilat | Nonsteroidal anti-inflammatory drugs | Aspirin |
|  | Benazepril |  | Ibuprofen |
|  | Accupril |  | ASA |
|  | Quinapril |  | Ketorolac |
|  | Zestril |  | Toradol |
|  | Fosinopril sodium |  | Celebrex |
|  | Zestoretic 20/25 |  | Mobic |
|  | Enalapril maleate |  | Celecoxib |
| Acyclovir | Acyclovir |  | Naproxen |
|  | Zovirax |  | Aspirin enteric coated |
| Allopurinol | Allopurinol |  | Motrin |
|  | Zyloprim |  | Indomethacin |
| Aminoglycoside | Gentamicin |  | Advil |
|  | Tobramycin |  | Meloxicam |
|  | Amikacin |  | Baby aspirin |
|  | Amikin |  | Aleve |
| Amphotericin | Amphotericin B |  | Voltaren |
|  | Fungizone |  | Nabumetone |
|  | Amphotericin B lipid complex |  | Excedrin |
|  | Amphotericin |  | Ecotrin |
|  | Abelcet |  | Darvon |
| Candesartan | Candesartan |  | Ketoprofen |
| Mycophenolic acid | Mycophenolate |  | Arthritis pain |
|  | Cellcept |  | Excedrin extra strength |
|  | Mycophenolate mofetil |  | Fiorinal |
| Cimetidine | Tagamet |  | Percodan |
| Ciprofloxacin | Ciprofloxacin |  | Asprin |
|  | Cipro |  | Oxaprozin |
|  | Ciprodex |  | Diclofenac sodium |
| Contrast | Optiray 320 |  | Indocin |
|  | Omnipaque 350 |  | Naprosyn |
|  | Visipaque |  | Piroxicam |
|  | Gadolinium |  | Diclofenac |
|  | IV contrast |  | Lodine |
|  | Radiopaque | Tacrolimus | Tacrolimus |
| Cyclosporine | Cyclosporine |  | Prograf |
|  | CSA | Tenofovir | Tenofovir disoproxil fumarate |
|  | Cyclosporine | Valsartan | Valsartan |
|  | Sandimmune |  | Diovan |
|  | Neural |  | Diovan hct 80/12.5 |
|  |  |  | Diovan hct 160/12.5 |
