# Supplementary material for: Genome-Wide Association Study of Serum Creatinine Levels during Vancomycin Therapy
Source: PLoS One. 2015 Jun 1;10(6):e0127791. doi: 10.1371/journal.pone.0127791 (PMC4452656; doi:10.1371/journal.pone.0127791)
Supplement: S2 Table — (DOCX) [file pone.0127791.s006.docx]

**S2 Table.** **Association of genotyped candidate chromosome 6 SNPs to peak creatinine in Marshfield validation cohort of 343 individuals.**

| SNP | Position | Risk Allele | RAF | β | SE | P |
| --- | --- | --- | --- | --- | --- | --- |
| rs12203935 | 121318076 | T | 0.09 | 0.17 | 0.06 | 0.003317 |
| rs12209104 | 121385438 | T | 0.09 | 0.17 | 0.06 | 0.003317 |
| rs9490288 | 121835186 | G | 0.36 | -0.09 | 0.03 | 0.01231 |
| rs938498 | 121354340 | C | 0.12 | -0.12 | 0.05 | 0.01719 |
| rs4946526 | 121294026 | A | 0.29 | -0.08 | 0.03 | 0.02143 |
| rs12529695 | 121776975 | A | 0.30 | 0.08 | 0.04 | 0.02295 |
| rs9375032 | 121827852 | T | 0.39 | 0.08 | 0.03 | 0.02474 |
| rs6922074 | 121302715 | T | 0.15 | -0.09 | 0.04 | 0.03322 |
| rs2389541 | 121815698 | C | 0.33 | -0.07 | 0.04 | 0.04562 |
| rs6926994 | 121286192 | G | 0.26 | -0.07 | 0.04 | 0.04625 |
| rs7747737 | 121286976 | T | 0.26 | -0.07 | 0.04 | 0.04625 |
| rs7747237 | 121286679 | A | 0.26 | -0.07 | 0.04 | 0.04762 |
| rs12211378 | 121836062 | G | 0.21 | -0.09 | 0.04 | 0.05323 |
| rs12207216 | 121326719 | A | 0.38 | -0.07 | 0.04 | 0.06533 |
| rs12212865 | 121812163 | T | 0.13 | 0.08 | 0.05 | 0.09824 |
| rs10456940 | 121805957 | C | 0.28 | -0.06 | 0.04 | 0.1025 |
| rs9482095 | 121281878 | A | 0.13 | -0.07 | 0.05 | 0.1098 |
| rs17083553 | 121775368 | T | 0.17 | 0.07 | 0.05 | 0.1193 |
| rs11154028 | 121823405 | C | 0.33 | -0.05 | 0.03 | 0.1205 |
| rs9401363 | 121376236 | G | 0.21 | -0.06 | 0.04 | 0.1351 |
| rs7767681 | 121397552 | C | 0.04 | -0.13 | 0.09 | 0.1382 |
| rs17652897 | 121794103 | A | 0.30 | -0.05 | 0.03 | 0.1382 |
| rs2222097 | 121393698 | C | 0.16 | -0.06 | 0.04 | 0.1552 |
| rs10499101 | 121262670 | C | 0.16 | -0.06 | 0.04 | 0.1581 |
| rs6928603 | 121789664 | G | 0.06 | -0.09 | 0.07 | 0.168 |
| rs17083598 | 121788169 | A | 0.07 | -0.09 | 0.06 | 0.1755 |
| rs9490287 | 121834711 | C | 0.15 | -0.06 | 0.04 | 0.1982 |
| rs6932373 | 121832689 | A | 0.43 | -0.04 | 0.03 | 0.2744 |
| rs7748279 | 121279946 | A | 0.09 | -0.07 | 0.07 | 0.3191 |
| rs7760976 | 121300015 | A | 0.12 | 0.05 | 0.06 | 0.3297 |
| rs6917641 | 121305804 | G | 0.11 | -0.05 | 0.05 | 0.3478 |
| rs1886249 | 121791852 | C | 0.16 | -0.04 | 0.04 | 0.3504 |
| rs4945671 | 121331758 | T | 0.15 | -0.04 | 0.05 | 0.3551 |
| rs3805787 | 121800606 | C | 0.22 | -0.03 | 0.04 | 0.3651 |
| rs1591826 | 121237497 | G | 0.15 | -0.04 | 0.04 | 0.3748 |
| rs9374979 | 121229038 | A | 0.11 | 0.05 | 0.05 | 0.399 |
| rs78232330 | 121190942 | G | 0.05 | 0.06 | 0.08 | 0.4465 |
| rs13193713 | 121796896 | T | 0.12 | -0.03 | 0.05 | 0.5075 |
| rs2255487 | 121232734 | C | 0.32 | -0.02 | 0.04 | 0.5519 |
| rs2789047 | 121231501 | A | 0.28 | -0.02 | 0.04 | 0.5713 |
| rs17083640 | 121818205 | A | 0.29 | -0.02 | 0.04 | 0.5748 |
| rs2817949 | 121232295 | G | 0.44 | -0.02 | 0.03 | 0.5751 |
| rs6931977 | 121832394 | C | 0.21 | 0.02 | 0.04 | 0.5953 |
| rs9385183 | 121391553 | A | 0.12 | 0.03 | 0.06 | 0.6217 |
| rs12527161 | 121236583 | T | 0.26 | -0.02 | 0.04 | 0.6342 |
| rs9374980 | 121233253 | T | 0.26 | -0.02 | 0.04 | 0.6404 |
| rs9490255 | 121788838 | G | 0.40 | -0.02 | 0.03 | 0.6437 |
| rs76951915 | 121192173 | C | 0.03 | 0.04 | 0.10 | 0.6538 |
| rs4552768 | 121272241 | A | 0.11 | -0.03 | 0.06 | 0.6608 |
| rs11968593 | 121391598 | T | 0.05 | -0.04 | 0.09 | 0.6795 |
| rs7755171 | 121363704 | A | 0.16 | -0.02 | 0.05 | 0.6817 |
| rs2789048 | 121232017 | T | 0.31 | -0.02 | 0.04 | 0.6872 |
| rs908458 | 121313864 | C | 0.05 | -0.04 | 0.10 | 0.7293 |
| rs17699863 | 121216700 | G | 0.07 | 0.02 | 0.08 | 0.7433 |
| rs7740324 | 121795537 | C | 0.29 | 0.01 | 0.04 | 0.7668 |
| rs2789049 | 121232236 | G | 0.32 | -0.01 | 0.04 | 0.7742 |
| rs9387915 | 121398635 | A | 0.16 | 0.01 | 0.05 | 0.7777 |
| rs9320773 | 121218196 | A | 0.25 | -0.01 | 0.04 | 0.8228 |
| rs2817948 | 121232552 | G | 0.33 | -0.01 | 0.04 | 0.8466 |
| rs6569208 | 121835805 | T | 0.37 | -0.01 | 0.03 | 0.8705 |
| rs2357969 | 121234500 | G | 0.26 | -0.01 | 0.04 | 0.8854 |
| rs77023002 | 121192067 | C | 0.04 | 0.00 | 0.11 | 0.9711 |
| rs2063878 | 121301914 | A | 0.22 | 0.00 | 0.04 | 0.9714 |
| rs78482893 | 121191397 | A | 0.04 | 0.00 | 0.11 | 0.9745 |
| rs2817966 | 121219850 | C | 0.05 | 0.00 | 0.08 | 0.9889 |

RAF – Risk Allele Frequency; SE – Standard Error
