# Supplementary material for: Genome-Wide Association Study of Serum Creatinine Levels during Vancomycin Therapy
Source: PLoS One. 2015 Jun 1;10(6):e0127791. doi: 10.1371/journal.pone.0127791 (PMC4452656; doi:10.1371/journal.pone.0127791)
Supplement: S3 Table — (DOCX) [file pone.0127791.s007.docx]

**S3 Table. Primary cohort demographics for the outcomes of vancomycin trough and vancomycin renal elimination rate constant, Ke.**

|  | Primary Cohort  (N=745) |
| --- | --- |
| Age (years)* | 52 (41-62) |
| Male^ | 429 (58%) |
| Weight (kg)* | 81 (67-97) |
| Height (m)* | 173 (165-180) |
| Vancomycin Dose (mg)* | 1000  (1000-1000) |
| Vancomycin Dosing Interval (h)* | 12 (8-12) |
| Vancomycin Trough (mcg/mL)* | 11 (7-16) |
| Baseline Creatinine (mg/dL)* | 0.7 (0.6-0.9) |
| Creatinine at Vancomycin Start (mg/dL)* | 0.8 (0.7-1.1) |
| 24-hour Loop Diuretic Dose (mg)* | 0 (0-0) |
| Loop Diuretic Exposure^ | 171 (23%) |
| Number of Non-loop Diuretics^  0  1 or more | 658 (88%)  87 (12%) |
| Number of Nephrotoxic Drugs^  0  1  2 or more | 288 (39%)  275 (37%)  182 (24%) |
| Peak Creatinine (mg/dL)* | 0.9 (0.8 – 1.3) |

*Median (interquartile range); ^N, %.
