# Supplementary material for: Genome-Wide Association Study of Serum Creatinine Levels during Vancomycin Therapy
Source: PLoS One. 2015 Jun 1;10(6):e0127791. doi: 10.1371/journal.pone.0127791 (PMC4452656; doi:10.1371/journal.pone.0127791)
Supplement: S4 Table — (DOCX) [file pone.0127791.s008.docx]

**S4 Table. Results for chromosome 1 and 5 SNP association with vancomycin trough in primary and validation cohorts.**

| Chr | SNP | Position | Risk Allele | Primary (N=745) | | | Marshfield (N=343) | | Meta β (SE) | Meta P |
| --- | --- | --- | --- | --- | --- | --- | --- | --- | --- | --- |
|  |  |  |  | RAF | β (SE) | P | β (SE) | P |  |  |
| 5 | rs12518285 | 84272817 | T | 0.27 | 0.27 (0.06) | 1.61x10^-6^ | 0.06 (0.05) | 0.23 | 0.15 (0.04) | 6x10^-5^ |
| 5 | rs10085144 | 84276133 | G | 0.27 | 0.27 (0.06) | 1.77x10^-6^ | 0.06 (0.05) | 0.25 | 0.15 (0.04) | 7x10^-5^ |
| 5 | rs62360865 | 84285547 | A | 0.25 | 0.26 (0.06) | 5.54x10^-6^ | 0.07 (0.05) | 0.16 | 0.15 (0.04) | 7x10^-5^ |
| 5 | rs9293392 | 84284722 | T | 0.26 | 0.26 (0.06) | 6.23x10^-6^ | 0.06 (0.05) | 0.2 | 0.15 (0.04) | 9x10^-5^ |
| 5 | rs10070381 | 84277367 | C | 0.26 | 0.26 (0.06) | 4.65x10^-6^ | 0.05 (0.05) | 0.26 | 0.14 (0.04) | 1x10^-4^ |
| 1 | rs35397194 | 220857144 | G | 0.09 | 0.34 (0.09) | 7.54x10^-5^ | 0.01 (0.09) | 0.89 | 0.18 (0.06) | 0.004 |
| 1 | rs3002142 | 220854685 | C | 0.12 | -0.31 (0.08) | 4.95x10^-5^ | -0.02 (0.07) | 0.80 | -0.13 (0.05) | 0.011 |
| 1 | rs3008608 | 220852621 | A | 0.12 | -0.31 (0.08) | 4.81x10^-5^ | -0.02 (0.07) | 0.80 | -0.13 (0.05) | 0.011 |
| 1 | rs3008604 | 220849819 | T | 0.12 | -0.31 (0.08) | 4.71x10^-5^ | -0.02 (0.07) | 0.74 | -0.13 (0.05) | 0.013 |
| 1 | rs3002143 | 220855477 | C | 0.12 | -0.31 (0.08) | 4.98x10^-5^ | -0.02 (0.07) | 0.72 | -0.13 (0.05) | 0.014 |
| 1 | rs35684750 | 220949025 | C | 0.16 | 0.27 (0.07) | 6.44x10^-5^ | -0.02 (0.06) | 0.77 | 0.11 (0.05) | 0.014 |
| 1 | rs35762933 | 220954530 | T | 0.15 | 0.29 (0.07) | 4.99x10^-5^ | -0.02 (0.06) | 0.79 | 0.12 (0.05) | 0.014 |
| 1 | rs3008607 | 220852315 | A | 0.12 | -0.31 (0.08) | 4.63x10^-5^ | -0.03 (0.07) | 0.68 | -0.13 (0.05) | 0.015 |
| 1 | rs3748631 | 220953211 | T | 0.12 | 0.31 (0.08) | 4.25x10^-5^ | -0.05 (0.07) | 0.47 | 0.12 (0.05) | 0.024 |
| 1 | rs3008610 | 220858530 | A | 0.12 | -0.31 (0.08) | 9.04x10^-5^ | -0.03 (0.07) | 0.62 | -0.11 (0.05) | 0.031 |
| 1 | rs3008634 | 220912034 | T | 0.12 | -0.29 (0.08) | 1.46x10^-4^ | -0.04 (0.07) | 0.55 | -0.11 (0.05) | 0.034 |
| 1 | rs1995152 | 220836216 | C | 0.16 | 0.09 (0.07) | 0.17 | 0.08 (0.06) | 0.16 | 0.09 (0.04) | 0.049 |
| 1 | rs10495197 | 220860323 | T | 0.09 | 0.37 (0.09) | 3.13x10^-5^ | -0.1 (0.08) | 0.24 | 0.12 (0.05) | 0.054 |
| 1 | rs17163303 | 220861741 | T | 0.09 | 0.37 (0.09) | 3.34x10^-5^ | -0.1 (0.08) | 0.24 | 0.12 (0.06) | 0.054 |
| 1 | rs74145511 | 222794864 | A | 0.09 | 0.37 (0.09) | 3.34x10^-5^ | -0.1 (0.08) | 0.22 | 0.11 (0.06) | 0.059 |
| 1 | rs75328711 | 222796127 | C | 0.09 | 0.37 (0.09) | 3.45x10^-5^ | -0.1 (0.08) | 0.22 | 0.12 (0.06) | 0.059 |
| 1 | rs142155704 | 222790560 | G | 0.05 | 0.55 (0.15) | 1.74x10^-4^ | -0.17 (0.14) | 0.22 | 0.18 (0.1) | 0.084 |
| 1 | rs1053316 | 220906461 | A | 0.12 | -0.29 (0.08) | 1.46x10^-4^ | -0.07 (0.06) | 0.28 | -0.08 (0.05) | 0.104 |
| 1 | rs2291834 | 220898918 | T | 0.27 | -0.14 (0.05) | 0.01 | -0.02 (0.05) | 0.66 | -0.05 (0.04) | 0.168 |
| 1 | rs35822937 | 220898633 | G | 0.15 | -0.04 (0.08) | 0.65 | 0.08 (0.05) | 0.13 | 0.05 (0.04) | 0.307 |
| 1 | rs17163429 | 220922038 | C | 0.21 | -0.12 (0.06) | 0.04 | 0.02 (0.05) | 0.71 | -0.04 (0.04) | 0.313 |
| 1 | rs904323 | 220856989 | A | 0.16 | 0.05 (0.07) | 0.42 | 0.01 (0.06) | 0.91 | 0.03 (0.04) | 0.539 |
| 1 | rs3002145 | 220873636 | T | 0.13 | 0 (0.07) | 0.96 | -0.03 (0.06) | 0.66 | -0.02 (0.05) | 0.709 |
| 1 | rs17011686 | 220911961 | G | 0.03 | -0.02 (0.13) | 0.89 | -0.02 (0.1) | 0.86 | -0.02 (0.08) | 0.82 |
| 1 | rs2378597 | 220930782 | T | 0.48 | -0.03 (0.05) | 0.59 | -0.01 (0.04) | 0.85 | -0.01 (0.03) | 0.837 |
| 1 | rs2088514 | 220897995 | G | 0.15 | -0.02 (0.07) | 0.76 | 0 (0.06) | 0.94 | -0.01 (0.04) | 0.888 |
| 1 | rs3002130 | 220838762 | C | 0.14 | -0.09 (0.07) | 0.22 | -0.05 (0.06) | 0.40 | 0.01 (0.05) | 0.895 |

RAF – Risk Allele Frequency; SE – Standard Error
